# Protective Effect of Pomegranate Seed Oil Against Acute Toxicity of Diazinon in Rat Kidney 

**Published:** 2013

**Authors:** Mohammad Taher Boroushaki, Delnia Arshadi, Hamideh Jalili-Rasti, Elham Asadpour, Azar Hosseini

**Affiliations:** a *Pharmacological Research Center of Medicinal Plants, Faculty of Medicine, Mashhad University of Medical Sciences, Mashhad, Iran. *; b *Deptartment of Pharmacology, Faculty of Medicine, Mashhad University of Medical Sciences, Mashhad, Iran. *

**Keywords:** Diazinon, Pomegranate seed oil, Oxidative stress, Lipid peroxidation, Kidney

## Abstract

Diazinon is an organophosphate which is extensively used in trade and agriculture. Due to its widespread application, its toxicity is common. Several studies have shown that organophosphates are able to induce oxidative stress by generating free radicals and depletion of endogenous antioxidants. Pomegranate seed oil (PSO) possesses anti-inflammatory and antioxidant effects. In this study, the effect of PSO was evaluated on diazinon-induced nephrotoxicity in rat.

Wistar male rats were randomly divided into four groups, 6 each. Group one received saline, 1 mL/kg, group 2 received diazinon 100 mg/kg. Groups 3 and 4 received PSO, 0.32 and 0.64 mg/kg, one hour before diazinon 100 mg/kg respectively. After 24 h, animals were anesthetized. Blood samples were taken out by cardiac puncture for measuring the level of serum urea and creatinine. 24 h urine samples were also collected for measuring glucose and protein concentration. The right kidney was removed and homogenized for measuring malondialdehyde and thiol content

Compare to control group, DIZ increased urea and serum creatinine, urinary glucose, and malondialdehyde, but did not modify significantly urinary protein and thiol content. In groups received PSO+ DIZ, serum creatinine, urinary glucose and MDA were significantly decreased.

DIZ induced acute nephrotoxicity and oxidative stress. Probably, increasing of serum creatinine and urinary glucose are appropriate markers for diagnosis of kidney damage. In addition increasing of MDA level emphasizes that DIZ plays role in pathogenesis of kidney via oxidative stress mechanism. PSO reduced DIZ toxicity by antioxidant activity.

## Introduction

Diazinon (DIZ) is a broad- spectrum organophosphate pesticide. Toxic effects of DIZ are due to inhibition of acetylcholinesterase activity, an enzyme needed for proper nervous system function. It has been widely used throughout the world with applications in agriculture and horticulture for controlling insects in crops and raising production efficiency (1). Studies have shown that DIZ has toxic effects on a range of cell lines such as blood cells, immune system, hepatocytes and renal cells (2, 3). In addition, many of these effects are resulting in excessive free radical production, consumption of tissue antioxidants and oxidative stress (4). However antioxidants can reduce the toxic effects of DIZ. It has been known that pomegranate seed oil has anti-inflammatory and antioxidant effects. Also studies have shown its kidney protective effect against various toxic materials (5). In this study, protective effect of PSO against DIZ-induced nephrotoxicity in rat was investigated.

## Experimental

Diazinon was purchased from Sigma, DTNB (2,2′-dinitro-5,5′-dithiodibenzoic acid), TBA (2-thiobarbituric acid), *n*-butanol, NaOH (sodium hydroxide), NaCl (sodium chloride), Na2EDTA (ethylenediaminetetraacetic acid disodium salt), Trizma base (Tris hydroxymethyl) aminomethane), phosphoric acid, HCl (hydrochloric acid), KCl (potassium chloride), ether, and TMP (tetramethoxypropane) were purchased from Merck (Darmstadt, Germany). PSO (*d *= 0.81 g/mL at 25°C) was a gift from Urom Narin Company. Production License No. 11616/12 (Uromeya, I. R. Iran). 

Adult male Wistar rats (Animal Breading Unit, School of Medicine, Mashhad, Iran), weighing 180–220 g were used for all experiments. Animals were housed in a pathogen-free facility on a 12 h light/dark schedule and with *ad libitum *access to food and water. All animal procedures were approved by the university ethics committee and were in compliance with national laws and with National Institutes of Health guidelines for the use and care of laboratory animals. After acclimatization, animals were randomly divided into four groups (6 each) and individually put in the metabolic cages. Group 1 (control group) was treated with saline (1 mL/kg). Group 2 received diazinon 100 mg/kg, and groups 3 and 4 received pomegranate seed oil 0.32 mg/kg and 0.64 mg/kg respectively, one hour before diazinon 100 mg/kg. All injections were carried out intraperitoneally.

After 24 h all animals were anesthetized under intraperitoneal injection of ketamine/xylazine (60 mg/kg and 6 mg/kg, respectively); blood samples were taken out by cardiac puncture for measuring the level of serum urea and creatinine. 24 h urine samples, for measuring glucose and protein concentration were also collected, before scarifying animals. The right kidney was removed, homogenized in cold KCl solution (1.5%, pH 7) to give a 10% homogenate suspension and used for measuring malondialdehyde (MDA) and thiol content. Urea concentration was determined colorimetrically using Autoanalyzer (Technicon RA-1000, England) and urea kit (Man Lab Company, Tehran, Iran). Creatinine concentration was measured by the Jaffe’s method (6).

Glucose concentration was estimated by the enzymatic assay (glucose oxidase) and protein concentration was measured by the turbidimetric method (7, 8). The lipid peroxidation level of the kidney tissue was measured as malondialdehyde, which is the end product of lipid peroxidation and reacts with TBA as a thiobarbituric acid reactive substance (TBARS) to produce a red-colored complex which has peak absorbance at 532 nm (9). Briefly, 3 mL phosphoric acid (1%) and 1 mL TBA (0.6%) were added to 0.5 mL of homogenate in a centrifuge tube and the mixture was heated for 45 min in a boiling water bath. After cooling, 4 mL of *n*-butanol was added to the mixture, vortexed for 1 min, and then centrifuged at 20,000 rpm for 20 min. The organic layer was transferred to a fresh tube and its absorbance was measured at 532 nm. The standard curve of MDA was constructed over the concentration range of 0-40 μM (10). Total SH groups were measured using DTNB as the reagent. This reagent reacts with the SH groups to produce a yellow-colored complex with peak absorbance at 412 nm. Briefly, 1 mL Tris–EDTA buffer (pH = 8.6) was added to 50 μL kidney homogenate in 2 mL cuvettes and sample absorbance was read at 412 nm against Tris–EDTA buffer alone (*A*1).Then 20 μL DTNB reagent (10 mM in methanol) was added to the mixture, and after 15 min (stored in laboratory temperature), the sample absorbance was read again (*A*2). The absorbance of DTNB reagent was also read as a blank (*B*). Total thiol concentration (mM) was calculated from the following equation (11): Total thiol concentration (mM) = (A_2_-A_1_-B) × 1.07/0.05 × 13.6

In a preliminary study, PSO alone did not significantly modify the biochemical parameters compare to the control group (data not shown).

*Statistical analysis*

Data were expressed as mean ± SEM. Statistical analysis was performed using one-way ANOVA

followed by Tukey-Kramer post-hoc test for multiple comparisons. The p-values < 0.05 were considered statistically significant.

## Results

No observable toxicity or any gross changes in kidney tissue of animals pretreated with PSO alone was observed.

*Concentration of serum creatinine in different treated groups*

**Figure 1:**
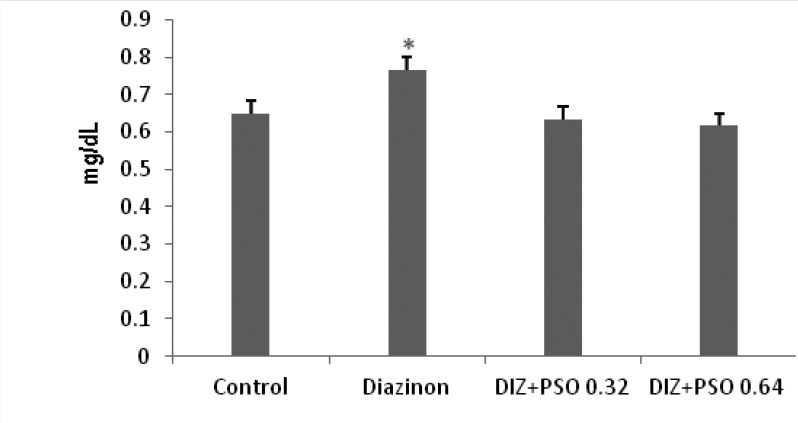
Concentration of serum creatinine in different treated groups. Values are mean ± SEM (n = 6). *p < 0.05 compare to control and PSO treated groups. DIZ: Diazinon, PSO: Pomegranate Seed Oil.

Data showed that DIZ significantly increased level of creatinine (p < 0.05) compare to control and PSO treated groups. There was no significant difference between control and PSO treated groups (Figure 1). 

*Concentration of serum urea in different treated groups*

**Figure 2:**
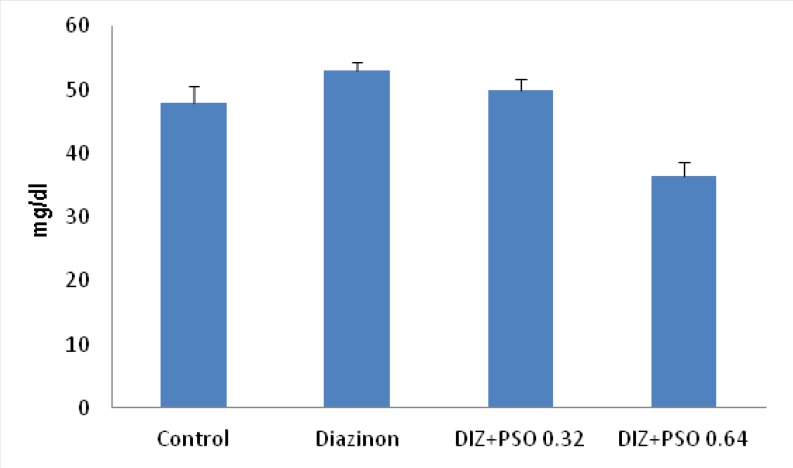
Concentration of serum urea in different treated groups.Values are mean ± SEM (n = 6). **p < 0.01 compare to control group,** p < 0.001compare to DIZ group. DIZ: Diazinon, PSO: Pomegranate Seed Oil.

Data showed that PSO, with 0.64mg/kg significantly decreased serum urea compare to control (p < 0.01) and DIZ (p < 0.001) treated groups. There was no significant difference between control and DIZ treated groups (Figure 2).

*Concentration of urinary glucose in different treated groups*

**Figure 3:**
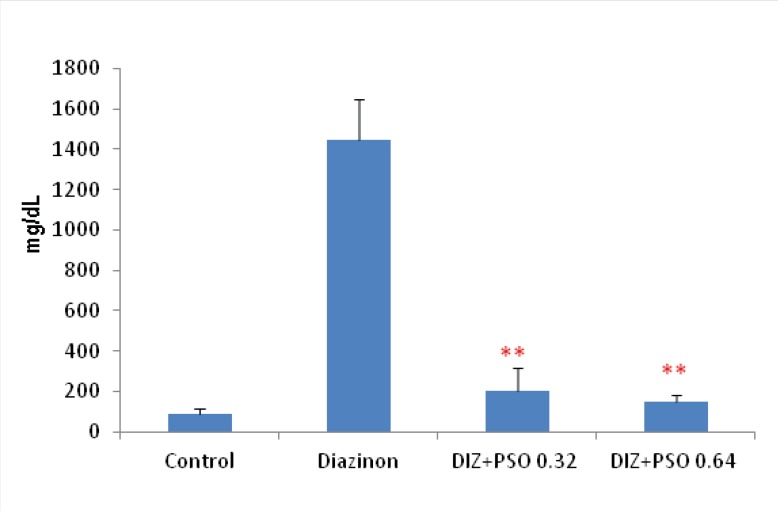
Concentration of urinary glucose in different treated groups. Values are mean±SEM (n=6). ** p < 0.01 compare to DIZ group ***p < 0.001 compare to control group. DIZ: Diazinon, PSO: Pomegranate Seed Oil

Results showed that in DIZ treated group concentration of urinary glucose was significantly increased compare to control group (p <0.001). PSO treated groups were significantly decreased urinary glucose compare to DIZ treated group (p < 0.01). There was no significant difference between control and PSO treated groups (Figure 3). 

*Concentration of urinary protein in different treated groups*

There were no significant differences among concentration of urinary protein in different treated groups.

*Concentration of MDA in different treated groups*

**Figure 4:**
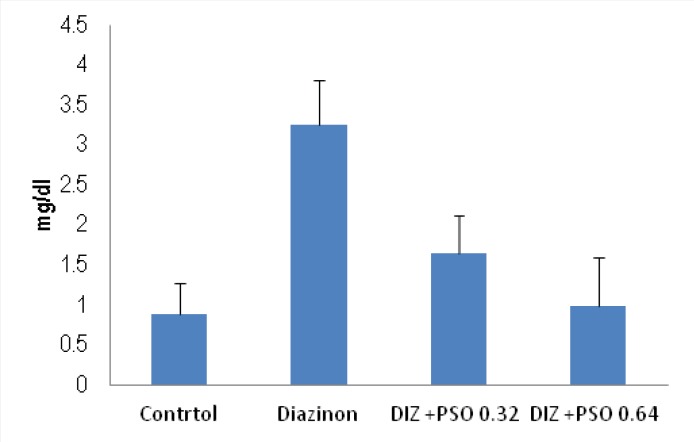
Concentration of MDA in different treated groups. Values are mean ± SEM (n = 6). * p < 0.05 compare to control and PSO 0.64mg/kg treated groups. DIZ: Diazinon, PSO: Pomegranate Seed Oil.

As shown there was significantly (p < 0.05) increase in concentration of MDA in DIZ treated compare to control and PSO 0.64mg/kg treated groups (Figure 4). 

*Concentration of thiol content in different treated groups*

There were no significant differences among concentration of total thiol in different treated groups.

## Discussion

Oxidative stress increases the production of free radicals and reduction of endogenous antioxidants. Free radicals have high activity and react with different molecules. However, they can disrupt the structure and function of proteins and enzymes (12). In addition, free radicals can lead to mutation and DNA damage (13). It is known that oxidative stress plays role in the pathogenesis of kidney toxicity of many toxins and drugs (14). DIZ is an organophosphate insecticide that has a lot of commercial and agricultural uses. It is also one of the most common causes of poisoning, therefore its effects on various organs is important. DIZ toxicity can be acute or chronic. Acute toxicity occurs in suicide or accidental ingestion, while chronic toxicity is due to long-term exposure (15). The kidney is responsible for elimination of active metabolites. DIZ metabolites are excreted via kidney. However, DIZ and its metabolites elimination through kidney are associated with kidney and tubular damage. This study indicated that DIZ has induced oxidative stress and kidney dysfunction, also acute toxicity of DIZ increases serum creatinine and urea. Concentration of creatinine is a marker of kidney function and its elevation is associated with kidney injury. Serum creatinine also shows kidney clearance. Therefore, increasing of creatinine is resulting of DIZ-induced kidney damage and clearance reduction. 

In a study, DIZ was given through gavage to rats, after 4 weeks some biochemical markers were evaluated. Results showed, serum creatinine and urea did not change. It could be due to low dose and oral method (16). In another study, DIZ was given orally for 8 weeks, urea and serum creatinine were increased (4). This is well agreed with our results. The present study has shown that PSO with doses of 0.32 and 0.64 mg / kg has reduced serum creatinine and urea levels in treated groups. 

It is believed that natural compounds and their derivatives represent a source of potential chemotherapeutic agents. Dietary supplementation with these products rich in antioxidants is associated with inhibition of toxicity of many chemicals (17). The results obtained in this study suggest that PSO has an overall protective effect against DIZ-induced nephrotoxicity in rat model. The observed protective effects can be attributed to the antioxidant properties of PSO that has been shown in our previous study (18). 

In this study, urinary glucose level was increased. Selectively, kidneys reabsorb urine glucose and prevent its excretion. Increasing of glucose in DIZ group shows that DIZ causes acute tubular damage and disturbs glucose uptake. In addition, urinary protein did not change. Whereas, serum proteins such as albumin have high molecular weight, it can be concluded that DIZ-induced kidney damage has not been enough to enter proteins into urine, but severely impairs glucose uptake. Two doses of PSO reduced urinary glucose in treated groups. This process shows that PSO restores glucose uptake and prevents its excretion. In fact, PSO reduces DIZ-induced tubular damage. 

The present study showed that acute dose of DIZ increased concentration of MDA but did not change total thiol contents of kidney hemogenates. MDA is a stable metabolite of the free radical-mediated lipid peroxidation cascade and it is known as a marker of oxidative stress. In this study PSO with dose of 0.64 mg / kg reduced MDA and inhibited DIZ-induced oxidative stress. These results clearly demonstrated that oxidative stress plays a role in DIZ toxicity and PSO reduced kidney toxicity of DIZ by antioxidant effects. Also, previous studies have shown that DIZ caused oxidative stress in kidney tissue of rat and increased MDA (19). These results well agreed with the results obtained in this study. Total thiol content was not increased significantly in this study, possibly depletion of thiol antioxidant factors may require more time and should be identified in sub-acute and chronic toxicities. Previous studies have been shown antioxidant effects of pomegranate seed oil (20-22). Pomegranate seed oil contains significant amounts of polyunsaturated fatty acids, which have antioxidant effects. Different studies have been shown that it has scavenger properties of free radicals and prevents lipid oxidation (23, 24). 

## Conclusion

In conclusion, the results of this study showed that PSO clearly attenuated DIZ-induced nephrotoxicity via; a) improving kidney function by reducing urinary glucose; b) reducing serum urea and creatinine; and c) decreasing MDA concentration, but explanation of these mechanisms need further investigations 
